# Prevalence of burnout syndrome and associated factors among nurses in Mogadishu, Somalia: a multicenter cross-sectional study

**DOI:** 10.3389/fpubh.2026.1951920

**Published:** 2026-10-01

**Authors:** Rahma Yusuf Haji Mohamud, Nur Adam Mohamed, Bakar Ali Odawa, Mohamed Farah Osman Hidig, Hassan Abdullahi Aden, Abdiladhif Mohamed Ali, Zerife Orhan, Serpil Doğan, Said Mohamed Mohamud, Suad Abdikarim Isse, Abukar Mahamed Osman

**Affiliations:** 1 Department of Health Services, Yardimeli Specialist Hospital, Mogadishu, Somalia; 2 Faculty of Medicine and Health Sciences, Jamhuriya University of Science and Technology (JUST), Mogadishu, Somalia; 3 Department of Psychiatry, Mogadishu Somali-Turkiye Recep Tayyip Erdogan Training and Research Hospital, Mogadishu, Somalia; 4 Department of Pediatrics, Hodan Hospital, Mogadishu, Somalia; 5 Department of Neurology, Kalkaal Hospital, Mogadishu, Somalia; 6 Department of Urology, Mogadishu Specialist Hospital, Mogadishu, Somalia; 7 Faculty of Medicine and Surgery, Somali National University, Mogadishu, Somalia; 8 Department of Neurology, Shaafi Hospital, Mogadishu, Somalia; 9 Department of Medical Services and Techniques, Vocational School of Health Services, Kahramanmaraş Sütçü Imam University, Kahramanmaraş, Türkiye; 10 Medical Microbiology Clinic, Necip Fazıl City Hospital, Kahramanmaraş, Türkiye; 11 Department of Medical Microbiology, Mogadishu Somali-Turkiye Recep Tayyip Erdogan Training and Research Hospital, Mogadishu, Somalia; 12 Department of Infection Prevention Control, Mogadishu Somali-Turkiye Recep Tayyip Erdogan Training and Research Hospital, Mogadishu, Somalia; 13 Department of Neurosurgery, Yardimeli Specialist Hospital, Mogadishu, Somalia

**Keywords:** associated factors, burnout syndrome, nurses, occupational health, prevalence, Somalia

## Abstract

**Background:**

Burnout is a major occupational health concern among nurses, with important implications for workforce well-being, quality of care, and patient safety. However, evidence on its prevalence and associated factors among nurses in Somalia remains limited. This study aimed to estimate the prevalence of burnout syndrome and identify associated factors among nurses in Mogadishu, Somalia.

**Methods:**

A multicenter cross-sectional study was conducted between 1 January and 28 February 2026 across five hospitals in Mogadishu. A total of 292 nurses completed an online self-administered questionnaire. Burnout was assessed using the Maslach Burnout Inventory–Human Services Survey (MBI-HSS) and defined as high emotional exhaustion (≥27), high depersonalization (≥13), and low personal accomplishment (≤31). Associations with sociodemographic, occupational, and psychosocial factors were examined using logistic regression. Results are presented as adjusted odds ratios (AORs) with 95% confidence intervals (CIs), with statistical significance set at *p* < 0.05.

**Results:**

A total of 292 nurses participated, of whom 18.2% (95% CI: 13.9–23.1) met the study definition of burnout. In the sociodemographic/occupational model, burnout was associated with mixed shift schedules, longer service duration, chronic illness, and high workload, with the strongest association observed among nurses with >10 years of service (AOR = 3.63, 95% CI: 1.40–9.40). In the psychosocial model, depressive symptoms, anxiety symptoms, poor sleep quality, and job dissatisfaction were associated with burnout, with depressive symptoms showing the strongest association (AOR = 5.85, 95% CI: 2.61–13.14).

**Conclusion:**

Nearly one in five nurses met the study definition of burnout, with several occupational, psychological, and health-related factors associated with burnout. These findings may inform workplace strategies to support nurses’ well-being, while longitudinal studies are needed to clarify temporal relationships.

## Introduction

Burnout is a work-related syndrome characterized by emotional exhaustion (EE), depersonalization (DP) (or cynicism), and a reduced sense of personal accomplishment (PA), arising from chronic occupational stress that has not been successfully managed (1, 2). It is widely recognized as a major occupational health concern, particularly in professions requiring sustained interpersonal engagement and emotional labor (1). The World Health Organization (WHO) classifies burnout in the International Classification of Diseases, 11th Revision (ICD-11), as an occupational phenomenon linked to workplace stress (2). This framing underscores burnout not only as an individual condition but also as a systemic issue shaped by working conditions, organizational structures, and resource constraints.

Globally, burnout has become a significant challenge among healthcare professionals, affecting workforce well-being, service quality, and patient safety (3–5). Evidence from systematic reviews in sub-Saharan Africa, Arab countries, and the Middle East report a high burden of burnout among healthcare workers, although prevalence estimates vary across settings and professions (6–8). Such differences may reflect variations in workload, healthcare infrastructure, staffing capacity, and psychosocial work environments. Burnout has been linked to reduced productivity, increased medical errors, poorer quality of care, and higher turnover intentions (3, 9).

Among healthcare professionals, nurses are particularly vulnerable to burnout due to their continuous patient interaction, emotional labor, shift work, and high levels of responsibility, often within resource-constrained and understaffed environments (10). Burnout among nurses is widespread, with meta-analytic evidence showing substantial levels of EE and reduced PA (11). This vulnerability is compounded by high workload, moral distress, workplace conflict, and limited organizational support (3, 11, 12). These pressures place nurses at heightened risk, with implications for their well-being, patient outcomes, and healthcare system performance (3).

The burden is particularly pronounced in low- and middle-income countries (LMICs) (12), especially in sub-Saharan Africa (6), where healthcare systems face substantial constraints. Regional evidence indicates high and variable burnout prevalence among healthcare workers, including nurses (6, 13, 14). This reflects workforce shortages, high patient-to-provider ratios, inadequate infrastructure, limited mental health support, and challenging working conditions (10, 11).

In Somalia, the healthcare system faces persistent challenges, including prolonged instability, limited infrastructure, and critical shortages of trained personnel, with healthcare delivery fragmented across public, private, and humanitarian sectors and services concentrated largely in urban centers (15, 16). Evidence from Mogadishu indicates that burnout is a significant concern among healthcare workers, with associations reported for poor sleep quality, psychological distress, and sociodemographic factors (13). However, empirical evidence remains limited, and existing studies have focused on mixed healthcare populations rather than nurses specifically (13). Given the distinct roles and stressors in nursing, findings from broader groups may not accurately reflect burnout among nurses. This gap is particularly relevant in a setting characterized by high workload, resource constraints, and evolving service demands. The Job Demands–Resources (JD-R) Model provides a useful framework for understanding burnout among nurses, proposing that burnout may develop when job demands exceed available resources. In the present study, high workload and mixed shift schedules were conceptualized as job demands, whereas social support and job satisfaction represented potential resources. Longer service duration and health- and recovery-related factors, including chronic illness, poor sleep, and psychological distress, were considered additional conditions that may influence exposure to demands or the capacity to cope with them. Accordingly, the JD-R framework informed the selection and interpretation of variables examined in relation to burnout (17, 18).

Previous research suggests that excessive workload and shift work may contribute to burnout through sustained occupational stress, inadequate recovery time, and depletion of cognitive and emotional resources (1, 19, 20). Longer service duration may reflect cumulative exposure to occupational demands and repeated workplace stressors, potentially increasing vulnerability to burnout over time (21, 22). Chronic health conditions may also increase susceptibility through persistent symptoms, fatigue, functional limitations, and the additional burden of managing health needs alongside demanding work responsibilities (23, 24). Sleep disturbances may further impair emotional regulation, cognitive functioning, and resilience to workplace stress, while psychological distress may diminish coping capacity and increase vulnerability to emotional exhaustion (25, 26). These mechanisms provide a plausible basis for examining occupational, sleep-related, and psychological factors in relation to burnout among nurses. Therefore, this study aimed to assess the prevalence of burnout syndrome and identify associated factors among nurses in Mogadishu, Somalia.

## Methods

### Study design and setting

This multicenter cross-sectional study was conducted between 1 January and 28 February 2026 across five hospitals in Mogadishu, Somalia. The participating institutions were Mogadishu Somali–Turkiye Recep Tayyip Erdogan Training and Research Hospital, Shaafi Hospital, Mogadishu Specialist Hospital, Hodan Hospital, and Kalkaal Hospital. These facilities represent both public and private healthcare sectors and provide general, emergency, and specialized services.

### Sample size and sampling procedure

The sample size was calculated using the single population proportion formula, as described by Charan: *n* = (z)^2^ * p * (1–p) / d^2^ (27), where *n* denotes the required sample size, Z = 1.96 corresponds to a 95% confidence level, and *d* = 0.05 represents the margin of error.

The estimated proportion (*p* = 49.2%) was obtained from a multicenter study conducted among nurses in Ethiopia (14), as it provided a population-specific estimate more directly comparable to the target study population. Although a local study from Somalia reported burnout prevalence among healthcare workers (13), it was not restricted to nurses and was therefore not used as the primary reference for parameter estimation.

This calculation yielded an initial sample size of 384. Given a total population of 602 nurses across the five participating hospitals, finite population correction yielded an adjusted sample size of approximately 235. To account for an anticipated 20% non-response rate, the adjusted sample size was divided by 0.80, resulting in a final target sample size of 294 participants.

Eligible nurses in the selected hospitals were invited to participate through an online self-administered questionnaire. A non-probability convenience sampling approach was employed, with 292 participants recruited during the study period. The sample size calculation was used primarily to establish a pragmatic recruitment target for prevalence estimation. Because participants were not selected using probability sampling, the finite population correction should not be interpreted as ensuring population-level statistical precision or representativeness. Furthermore, the prevalence-based calculation was not designed specifically to establish statistical power for the multivariable logistic regression analysis.

### Study population and recruitment

Nurses employed at the five selected hospitals during the data-collection period were invited to participate if they met the predefined inclusion criteria. Eligibility was restricted to nursing staff to align with the study objective of assessing burnout within this professional group. Recruitment was coordinated by designated research team members at each hospital, who initially contacted nursing supervisors or unit heads either in person or by telephone. During these interactions, the study objectives and procedures were explained, and support for facilitating staff participation was sought.

Detailed study information and a secure Google Forms link to the self-administered questionnaire were subsequently provided for distribution to eligible nurses via established WhatsApp groups within each hospital. WhatsApp-based recruitment was selected because it is a widely used communication platform among healthcare professionals in Somalia and enabled efficient recruitment of nurses across multiple hospitals while minimizing logistical constraints. To maximize response rates and minimize incomplete submissions, periodic reminders were issued throughout the data-collection period.

### Inclusion and exclusion criteria

Participants were eligible if they were nurses aged ≥18 years and actively engaged in professional duties during the study period. Only those who provided informed consent and completed the online questionnaire were included in the final analysis. Exclusion criteria included absence from work for more than four weeks due to leave or suspension, lack of formal employment at a participating hospital, inability to provide informed consent, or inability to read and understand the English-language questionnaire. English comprehension was assessed by self-report as part of the online consent process before participants proceeded to the questionnaire.

### Study variables and instruments

#### Dependent variable

The primary outcome was burnout syndrome, assessed using the Maslach Burnout Inventory–Human Services Survey (MBI-HSS) (28). Participants meeting the predefined three-dimensional criterion described below were classified as experiencing burnout.

#### Independent variables

A broad range of variables was assessed across sociodemographic, occupational, behavioral, and psychosocial domains. Socio-demographic factors included age, biological sex, marital status, monthly income, education status, and presence of chronic medical condition. Occupational variables comprised job title, institutional affiliation, work shift, work hours per day, workload, and years of professional experience. Behavioral factors included physical activity and current use of psychoactive substance. Psychosocial measures encompassed job satisfaction, symptoms of depression and anxiety, sleep quality, and perceived social support. These variables were analyzed to identify factors associated with burnout syndrome among nurses.

The presence of chronic medical conditions was assessed using a single self-report item and categorized as yes/no. Participants reporting any diagnosed long-term condition (e.g., hypertension, diabetes, asthma, or cardiovascular disease) were classified as having a chronic illness (29).

Physical activity was assessed using a single self-report item and categorized dichotomously (yes/no) based on adherence to World Health Organization recommendations. Participants were classified as physically active if they reported at least 150 min of moderate-intensity activity, 75 min of vigorous-intensity activity, or an equivalent combination per week (30).

Psychoactive substance use was assessed using a self-report item covering alcohol consumption, cigarette smoking, khat chewing, hashish use, and other substances. Current use was defined as any use within the past 30 days and coded as a binary variable (yes/no) (31).

Workload was assessed using a single self-report item and categorized dichotomously (yes/no). Participants who reported experiencing a high workload—such as managing a large number of patients, time pressure, or frequent task overload—were classified as having a high workload, while those who did not report such conditions were categorized as not having a high workload (32).

### Data collection tools

Data were collected using self-administered instruments distributed electronically to nurses via WhatsApp groups. The survey included a structured questionnaire and validated psychometric scales, including the MBI-HSS (28), the Patient Health Questionnaire-2 (PHQ-2), the Generalized Anxiety Disorder Scale-2 (GAD-2), the Single-Item Sleep Quality Scale (SQS), a single-item measure of job satisfaction, and the Oslo Social Support Scale-3 (OSSS-3). The questionnaire was developed following a comprehensive review of relevant literature (13, 14, 29) to capture socio-demographic, occupational, behavioral, and psychosocial variables. Except for age, all variables were categorical and assessed using closed-ended response formats.

#### Burnout syndrome

Burnout was assessed using the 22-item MBI-HSS, comprising three dimensions: EE (9 items), DP (5 items), and PA (8 items) (28). Items are rated on a seven-point frequency scale ranging from 0 (“never”) to 6 (“every day”). Burnout was operationally defined as the concurrent presence of high EE (≥27), high DP (≥13), and low PA (≤31) (13, 14; Table 1). The original validation study demonstrated good internal consistency, with Cronbach’s α coefficients of 0.89 for EE, 0.77 for DP, and 0.74 for PA (28).

**Table 1:** Classification of MBI-HSS subscale scores.

Category	Emotional exhaustion	Depersonalization	Personal accomplishment
Low	≤ 16	≤ 6	≤ 31
Moderate	17–26	7–12	32–38
High	≥ 27	≥ 13	≥ 39

Higher EE and DP scores indicate greater burnout, whereas lower PA scores indicate greater burnout.

#### Depression symptoms

Depressive symptoms were assessed using the PHQ-2, a brief screening tool derived from the first two items of the PHQ-9 assessing (1) diminished interest or pleasure in activities and (2) depressed mood or hopelessness. Each item is scored from 0 to 3, yielding a total score of 0–6, with scores ≥3 indicating probable depression (33). The PHQ-2 has demonstrated good diagnostic validity across clinical settings (33, 34). In the original validation study, a cut-off score of ≥3 showed 83% sensitivity and 92% specificity for major depression (35).

#### Anxiety symptoms

Anxiety symptoms were measured using the GAD-2, a brief screening tool derived from the first two items of the GAD-7 assessing (1) nervousness or feeling on edge and (2) difficulty controlling worry. Each item is scored from 0 to 3, yielding a total score of 0–6, with scores ≥3 indicating probable anxiety (33). The GAD-2 has demonstrated good psychometric properties and diagnostic performance across clinical populations (33, 34, 36), with a primary-care validation study reporting areas under the receiver operating characteristic curve ranging from 0.80 to 0.91 across anxiety disorders (36).

#### Sleep quality

Sleep quality was assessed using the Single-Item SQS, a validated measure developed by Snyder and colleagues (37). Participants rated their overall sleep quality over the past week on a 0–10 scale, with 0 indicating very poor and 10 indicating excellent sleep. Scores ≤6 were classified as poor sleep quality, whereas scores >6 indicated good sleep (38). The SQS is a brief and reliable measure of subjective sleep quality (37). The SQS has demonstrated concurrent and construct validity against established sleep-quality measures, as well as acceptable test–retest reliability in its original psychometric evaluation (37).

#### Job satisfaction

Job satisfaction was assessed using a single-item global measure (39). Participants rated their overall job satisfaction on a four-point scale ranging from very satisfied to very dissatisfied. For analysis, responses were dichotomized as satisfied (very or somewhat satisfied) or dissatisfied (somewhat or very dissatisfied). Previous studies have supported the reliability and validity of single-item measures of overall job satisfaction (40).

#### Social support

Perceived social support was assessed using the OSSS-3, developed by Dalgard (41). Total scores range from 3 to 14 and are categorized as poor (3–8), moderate (9–11), or strong (12–14) support. The OSSS-3 is a brief, reliable instrument widely used in psychological and public health research (42, 43). The OSSS-3 has demonstrated acceptable internal consistency (Cronbach’s α = 0.64) and a unidimensional factor structure in a population-based validation study (44).

Internal consistency estimates for the multi-item instruments and subscales calculated in the current sample are presented in Supplementary Table S1.

Although these instruments have not been formally validated among Somali nurses, they were selected due to their widespread use, strong psychometric properties, and demonstrated validity across diverse cultural and occupational contexts, including low- and middle-income countries (14, 45). English is the primary language of higher education and professional healthcare training in Somalia, and all participants had completed their academic and professional education in English. In line with the exclusion criteria, individuals with insufficient English proficiency were excluded to ensure consistent comprehension of the questionnaire items. Accordingly, the original English versions of the MBI-HSS, PHQ-2, GAD-2, SQS, job satisfaction scale, and OSSS-3 were administered without translation, and standard cut-off values were applied in accordance with original validation studies and prior literature to ensure comparability.

Prior to data collection, the instruments were pilot-tested among 20 nurses from the target population to assess clarity, comprehension, and contextual relevance. No difficulties in understanding or responding to the items were reported, supporting their suitability in this population. However, the lack of formal translation and cross-cultural adaptation may limit the generalizability of the findings, particularly among individuals with lower English proficiency.

### Data collection procedures

Data were collected using self-administered questionnaires distributed electronically via WhatsApp and completed through Google Forms. The survey link was shared through official professional WhatsApp groups and personal communication channels to ensure broad access among eligible nurses. To prevent item-level missing data, all questionnaire items were set as mandatory, and each participant was allowed to submit the questionnaire only once.

### Data management and statistical analysis

Following data collection, responses were exported to Microsoft Excel for initial screening and cleaning. Categorical variables were numerically coded (e.g., yes/no as 1/0) to facilitate analysis. The cleaned dataset was then imported into IBM SPSS Statistics version 27 (IBM Corp., Armonk, NY, USA) for statistical analysis.

Descriptive statistics summarized socio-demographic, behavioral, occupational, and psychosocial characteristics. The prevalence of burnout syndrome was estimated with 95% confidence intervals (CIs) calculated using the Clopper–Pearson exact method (46).

Associations between the study variables and burnout syndrome were examined using bivariable and multivariable logistic regression analyses. Given the relatively limited number of burnout events and the number of candidate variables, separate multivariable logistic regression models were fitted for sociodemographic/occupational and psychosocial factors to reduce model complexity and the risk of overfitting. Variables with an overall bivariable *p*-value ≤0.25 were considered for inclusion in the multivariable model (47). Model fit and performance were evaluated using complementary indicators. The Omnibus Test of Model Coefficients assessed overall model significance, while the Hosmer–Lemeshow goodness-of-fit test examined calibration, with a non-significant result indicating acceptable fit (48). Nagelkerke pseudo-R^2^ was reported as an index of model explanatory performance. Discriminative performance was evaluated using the area under the receiver operating characteristic (ROC) curve (AUC). Multicollinearity was examined using variance inflation factor (VIF) and tolerance values, with VIF < 10 and tolerance >0.1 indicating acceptable levels. Statistical significance was set at *p* < 0.05, and results were reported as adjusted odds ratios (AORs) with 95% CIs.

### Ethical considerations

Ethical approval was obtained from the Institutional Review Board of Mogadishu Somali–Turkiye Recep Tayyip Erdogan Training and Research Hospital (Ref. No.: MSTH/24069). The study was conducted in accordance with the ethical principles of the Declaration of Helsinki. Participants were provided with detailed information regarding the study objectives, procedures, and their rights as research participants. Electronic informed consent was obtained through the anonymous online survey platform before participants proceeded to the questionnaire. Participation was voluntary, and participants could withdraw at any time before submission without penalty. Participants were also informed that, due to the anonymous nature of data collection, responses could not be withdrawn or deleted once submitted.

To ensure confidentiality, no personally identifiable information was collected. All responses were anonymized and securely stored throughout the study period.

## Results

### Socio-demographic and behavioral characteristics

Of the target sample of 294 nurses, 292 participants were recruited and included in the analysis. There were no item-level missing data among the analyzed participants. The majority were aged 18–29 years (73.6%) and more than two-thirds were female (67.8%). Most participants were unmarried (66.4%) and had a monthly family income of ≤600 USD (73.6%). Over half held a bachelor’s degree (52.4%), while a substantial proportion had a master’s degree or higher (39.4%). Most nurses reported no chronic medical conditions (79.5%) and engaged in physical activity (92.1%), and nearly three-quarters reported no current substance use (74.7%). The socio-demographic and behavioral characteristics of the participants are presented in Table 2.

**Table 2:** Socio-demographic and behavioral characteristics of the participants (*n* = 292).

Variable	Category	Frequency	Percentage
Age	18–29 years	215	73.6
	≥ 30 years	77	26.4
Biological sex	Male	94	32.2
	Female	198	67.8
Marital status	Unmarried	194	66.4
	Married	98	33.6
Monthly income	≤600 USD	215	73.6
	>600 USD	77	26.4
Education status	Diploma	24	8.2
	Bachelor degree	153	52.4
	Master’s and above	115	39.4
Chronic medical condition	Yes	60	20.5
	No	232	79.5
Physical activity	Yes	269	92.1
	No	23	7.9
Current substance use	Yes	74	25.3
	No	218	74.7

### Psychosocial and occupational characteristics

Among the 292 nurses, the majority were registered nurses (75.7%). Mixed shifts were the most common work schedule (45.9%), and over half of the participants worked ≤10 h per day (51.7%) and had less than five years of experience (56.2%). Most nurses were satisfied with their jobs (88.4%), although a high workload was reported by 68.8%. Regarding mental health, 26.7% of participants reported depressive symptoms and 22.3% reported anxiety symptoms. Poor sleep quality was observed in 56.5% of participants, and over half reported poor social support (51.4%). Detailed characteristics are presented in Table 3.

**Table 3:** Psychosocial and occupational characteristics of the participants (*n* = 292).

Variables	Categories	Frequency	Percentage
Job title	Nurse administrator	71	24.3
	Registered nurse (RN)	221	75.7
Work shift	Regular day shift	111	38.0
	Regular night shift	47	16.1
	Mixed shifts	134	45.9
Work hours per day	≤ 10 h	151	51.7
	> 10 h	141	48.3
Years of experience	< 5 years	164	56.2
	5–10 years	101	34.6
	> 10 years	27	9.2
Institutional affiliation	Erdogan hospital	149	51.0
	Hodan hospital	45	15.4
	Shaafi hospital	38	13.0
	Kalkaal hospital	32	11.0
	Mogadishu specialist hospital	28	9.6
Job satisfaction	Satisfied	258	88.4
	Dissatisfied	34	11.6
Workload	Yes	201	68.8
	No	91	31.2
Depression symptoms	Absent	214	73.3
	Present	78	26.7
Anxiety symptoms	Absent	227	77.7
	Present	65	22.3
Sleep quality	Poor	165	56.5
	Good	127	43.5
Social support	Poor	150	51.4
	Moderate	99	33.9
	High	43	14.7

### Prevalence of burnout syndrome among nurses

Among the 292 nurses surveyed, burnout subscales demonstrated varying levels of severity across dimensions. EE was low in 46.2% of participants, moderate in 24.0%, and high in 29.8%. DP showed a higher burden, with 41.4% reporting high levels, compared with 26.7 and 31.8% reporting moderate and low levels, respectively. PA was predominantly low (70.5%), with smaller proportions reporting moderate (13.4%) and high (16.1%) levels. Overall, 18.2% (95% CI: 13.9–23.1) of participants met the criteria for burnout syndrome. Notably, high depersonalization and low personal accomplishment were prominent features. Detailed results are presented in Table 4.

**Table 4:** Prevalence of burnout syndrome among nurses (*n* = 292).

Burnout measure	Category	Frequency	Percentage
EE subscale	High	87	29.8
	Moderate	70	24.0
	Low	135	46.2
DP subscale	High	121	41.4
	Moderate	78	26.7
	Low	93	31.8
PA subscale	High	47	16.1
	Moderate	39	13.4
	Low	206	70.5
Overall BOS status	Absent	239	81.8
	Present	53	18.2

EE, emotional exhaustion; DP, depersonalization; PA, personal accomplishment; BOS, burnout syndrome. Higher EE and DP scores indicate greater burnout, whereas lower PA scores indicate greater burnout.

### Socio-demographic and work-related factors associated with burnout syndrome

In bivariable logistic regression analyses, monthly income, work shift, service duration, chronic illness, and workload met the predefined criterion (*p* ≤ 0.25) for inclusion in the multivariable model; the remaining variables did not meet this criterion. After adjustment within the sociodemographic/occupational multivariable model, mixed shifts, service duration >10 years, chronic illness, and workload remained associated with burnout syndrome (*p* < 0.05), whereas monthly income was not statistically significant. The logistic regression model demonstrated good overall fit, with a statistically significant Omnibus Test of Model Coefficients (*χ^2^* (7) = 44.550, *p* < 0.001), indicating that the included variables collectively distinguished between nurses with and without burnout syndrome. The Hosmer–Lemeshow test was non-significant (*χ^2^* (8) = 6.114, *p* = 0.634), supporting adequate model calibration. The model had a Nagelkerke R^2^ of 0.231 and demonstrated acceptable discrimination, with an AUC of 0.776 (95% CI: 0.705–0.847, *p* < 0.001). Multicollinearity was not a concern, as indicated by VIF values <2 and tolerance values >0.9.

Nurses working mixed day–night shifts had higher odds of burnout syndrome compared with those on fixed schedules (AOR = 2.40, 95% CI: 1.13–5.12). Longer service duration (>10 years) was also associated with higher odds of burnout after adjustment within the sociodemographic/occupational model (AOR = 3.63, 95% CI: 1.40–9.40). Chronic illness (AOR = 3.34, 95% CI: 1.66–6.72) and high workload (AOR = 2.80, 95% CI: 1.16–6.73) were likewise associated with burnout within this model. Although these associations were statistically significant, the relatively wide confidence intervals for some estimates indicate uncertainty in their magnitude and warrant cautious interpretation. These findings are presented in Table 5.

**Table 5:** Logistic regression analyses of the socio-demographic and work-related factors associated with burnout syndrome (*n* = 292).

Variable	Category	BOS status		COR (95% CI)	AOR (95% CI)	*p*-value
		Present *n* (%)	Absent *n* (%)			
Monthly income	≤600 USD	43 (20.0)	172 (80.0)	1.68 (0.80–3.52)	2.06 (0.90–4.70)	0.086
	> 600 USD	10 (13.0)	67 (87.0)	1	1	
Work shift	Regular day shift	13 (11.7)	98 (88.3)	1	1	
	Regular night shift	7 (14.9)	40 (85.1)	1.32 (0.49–3.55)	1.36 (0.47–3.92)	0.572
	Mixed shifts	33 (24.6)	101 (75.4)	2.46 (1.22–4.96)	2.40 (1.13–5.11)	0.023*
Service duration	<5 years	31 (18.9)	133 (81.1)	1	1	
	5–10 years	10 (9.9)	91 (90.1)	0.47 (0.22–1.01)	0.52 (0.23–1.15)	0.107
	>10 years	12 (44.4)	15 (55.6)	3.43 (1.46–8.06)	3.63 (1.40–9.40)	0.008*
Chronic medical condition	Yes	22 (36.7)	38 (63.3)	3.75 (1.97–7.17)	3.34 (1.66–6.72)	<0.001*
	No	31 (13.4)	201 (86.6)	1	1	
Workload	Yes	46 (22.9)	155 (77.1)	3.56 (1.54–8.24)	2.80 (1.16–6.73)	0.022*
	No	7 (7.7)	84 (92.3)	1	1	

*p* < 0.05 was considered statistically significant; significant values are marked with an asterisk (*), COR: Crude odd ratio; AOR, Adjusted odd ratio; 1, reference categories, CI, Confidence interval.

### Psychosocial factors associated with burnout syndrome

In bivariable logistic regression analyses, depressive symptoms, anxiety symptoms, social support, sleep quality, and job satisfaction met the predefined criterion (*p* ≤ 0.25) for inclusion in the multivariable model. After adjustment within the psychosocial multivariable model, depressive symptoms, anxiety symptoms, poor sleep quality, and job dissatisfaction remained associated with burnout syndrome (*p* < 0.05), whereas social support was not statistically significant. The logistic regression model demonstrated good overall fit, with a statistically significant Omnibus Test of Model Coefficients (*χ^2^* (6) = 79.479, *p* < 0.001), indicating that the included variables collectively distinguished between nurses with and without burnout. The Hosmer–Lemeshow test was non-significant (*χ^2^* (7) = 3.044, *p* = 0.881), supporting adequate model calibration. The model had a Nagelkerke R^2^ of 0.389 and demonstrated good discrimination, with an AUC of 0.850 (95% CI: 0.794–0.904, *p* < 0.001). Multicollinearity was not a concern, as VIF values were <2 and tolerance values >0.7.

Nurses with depressive symptoms had higher odds of burnout syndrome compared with those without (AOR = 5.85, 95% CI: 2.61–13.14). Anxiety symptoms (AOR = 3.27, 95% CI: 1.50–7.17), poor sleep quality (AOR = 3.01, 95% CI: 1.35–6.72), and job dissatisfaction (AOR = 3.10, 95% CI: 1.17–8.24) were likewise associated with burnout within the psychosocial model. Although these associations were statistically significant, the relatively wide confidence intervals indicate uncertainty in the magnitude of some estimates and warrant cautious interpretation. These findings are presented in Table 6.

**Table 6:** Logistic regression analyses of the psychosocial factors associated with burnout syndrome (*n* = 292).

Variable	Category	BOS status		COR (95% CI)	AOR (95% CI)	*p*-value
		Present *n* (%)	Absent *n* (%)			
Depression symptoms	Absent	20 (9.3)	194 (90.7)	1	1	
	Present	33 (42.3)	45 (57.7)	7.11 (3.74–13.53)	5.85 (2.61–13.14)	<0.001*
Anxiety symptoms	Absent	24 (10.6)	203 (89.4)	1	1	
	Present	29 (44.6)	36 (55.4)	6.81 (3.57–13.01)	3.27 (1.49–7.17)	0.003*
Social support	Low	41 (27.3)	109 (72.7)	3.67 (1.23–10.91)	2.36 (0.71–7.86)	0.162
	Moderate	8 (8.1)	91 (91.9)	0.86 (0.24–3.01)	0.40 (0.10–1.65)	0.205
	High	4 (9.3)	39 (90.7)	1	1	
Sleep quality	Poor	38 (23.0)	127 (77.0)	2.23 (1.17–4.28)	3.01 (1.35–6.72)	0.007*
	Good	15 (11.8)	112 (88.2)	1	1	
Job satisfaction	Satisfied	40 (15.5)	218 (84.5)	1	1	
	Dissatisfied	13 (38.2)	21 (61.8)	3.37 (1.56–7.28)	3.10 (1.17–8.24)	0.023*

*p* < 0.05 was considered statistically significant; significant values are marked with an asterisk (*), COR, Crude odd ratio; AOR, Adjusted odd ratio; 1, reference categories; CI, Confidence interval.

## Discussion

The present study provides important insight into burnout and its associated factors among nurses in Mogadishu, with nearly one in five participants (18.2%) meeting the study definition of burnout. Mixed shift schedules, longer service duration, and high workload were associated with higher odds of burnout within the occupational model, while job dissatisfaction was associated with higher odds of burnout within the psychosocial model. Chronic medical conditions and psychological distress—particularly depressive and anxiety symptoms, as well as poor sleep quality—were also associated with increased vulnerability. Together, these findings highlight the interplay between occupational, psychological, and health-related influences on burnout, underscoring the need for multidimensional interventions in this setting.

Burnout syndrome is a work-related psychological condition arising from chronic occupational stress and is widely recognized as a significant concern among healthcare workers, particularly nurses exposed to high workloads, shift work, and sustained emotional demands. The prevalence observed in this study is broadly consistent with estimates reported in comparable settings, including Iran (49), Brazil (50), and Ethiopia (51), as well as a systematic review and meta-analysis from Spain (52). However, it remains lower than estimates from other Ethiopian studies (14, 32) and Saudi Arabia (45), while exceeding those reported in Taiwan (53) and global pooled estimates (54). Such variation may reflect differences in healthcare systems, study populations, measurement approaches, and operational definitions. Notably, the three-dimensional criterion used in the present study may yield lower prevalence estimates than definitions based on fewer MBI dimensions. This methodological variability is illustrated by a recent study among university staff in Cameroon, which reported a burnout prevalence of 59.6% using a more inclusive definition in which a high level in at least one MBI dimension was sufficient for classification (55). Accordingly, prevalence comparisons across studies using different operational definitions should be interpreted cautiously.

Mixed shift schedules have consistently been linked to higher burnout levels among nurses, reflecting the cumulative strain of irregular and unpredictable work patterns (56, 57). Nurses working alternating day–night shifts in this study showed higher odds of burnout compared with those on fixed schedules, aligning with findings from Iran (49), Jordan (58), and Palestine (56). Evidence from systematic reviews further indicates that mixed shifts are particularly associated with emotional exhaustion, driven by frequent night duties, inadequate recovery time, and organizational stressors such as staffing shortages (57). Disruption of circadian rhythms appears central to this relationship, as shift work—particularly night rotations—impairs sleep quality and contributes to chronic fatigue and emotional exhaustion (25). Irregular schedules may also disrupt hormonal regulation, including cortisol and melatonin secretion, further influencing stress responses and mood (59). In parallel, work–life imbalance and reduced recovery opportunities may intensify psychological strain (60).

High workload and increased patient demands are consistently associated with elevated burnout levels among healthcare professionals (61). Nurses exposed to high workload in this study showed higher odds of burnout than those reporting manageable workloads, consistent with findings from Indonesia (62) and Ethiopia (63). Similarly, a systematic review by Ramírez-Elvira et al. identified adverse working conditions, particularly excessive workload, as key contributors to burnout among nurses in intensive care settings (64). This pattern likely reflects interconnected pathways. High workload has been linked to greater physical and emotional strain, reduced opportunities for rest and recovery, and increased emotional exhaustion (1, 19). Similarly, high job demands have been associated with depletion of cognitive and emotional resources, impaired coping capacity, and lower resilience to occupational stress (20).

In addition, heavy workload frequently co-occurs with organizational challenges such as inadequate staffing, limited managerial support, and time pressure, which have also been associated with higher levels of psychological strain among nurses (65). These factors may help explain the association between high workload and burnout observed in the present study.

Longer professional service duration has been associated with an increased risk of burnout among nurses (66). Nurses with more than 10 years of experience in this study showed higher odds of burnout than those with fewer years of service, consistent with findings from Uganda (21). This pattern likely reflects cumulative occupational exposure. Prolonged exposure to workplace stressors may lead to chronic physical and emotional strain, increasing susceptibility to emotional exhaustion and reduced resilience (21, 22). Extended service may also involve repeated exposure to high workload, shift work, and emotionally demanding patient care, contributing to depersonalization and reduced professional fulfillment (21). Sustained workplace stress and long-term job demands have likewise been identified as key contributors to burnout among nurses (67). Overall, these factors offer plausible occupational and psychological explanations for the higher likelihood of burnout among nurses with extended service duration.

Chronic illness has been associated with higher levels of occupational burnout among working populations in demanding environments (68, 69). Nurses with chronic illness in this study showed higher odds of burnout than those without such conditions, consistent with prior evidence linking poorer physical health to increased burnout risk (63, 70). This relationship may be bidirectional, as chronic disease may increase vulnerability to burnout, while burnout may contribute to physical and psychological health problems (23). Potential pathways include the burden of persistent symptoms, fatigue, sleep disturbances, and reduced functional capacity (23), which may heighten susceptibility to emotional exhaustion and impaired work performance. Managing chronic conditions alongside demanding work roles may further increase stress perception, weaken coping capacity, and reduce resilience to workplace stressors (24). In addition, organizational pressures—such as understaffing, long working hours, and limited recovery opportunities—may compound psychological strain among affected nurses. Together, these factors suggest that interacting biological, psychological, and occupational influences may contribute to increased burnout vulnerability among nurses with chronic illness.

Low job satisfaction has been consistently associated with elevated burnout among healthcare professionals, particularly nurses (71, 72). Nurses reporting job dissatisfaction in this study showed higher odds of burnout than those satisfied with their work, consistent with findings from Ethiopia (14), China (73), and Syria (74), as well as a global systematic review and meta-analysis of intensive care unit nurses (72). Job dissatisfaction may reflect unmet professional expectations, inadequate recognition, and limited organizational support—factors linked to emotional exhaustion and reduced motivation among healthcare workers (1). In addition, dissatisfaction with work environments, including poor leadership, inadequate staffing, limited autonomy, and reduced schedule flexibility, may impair coping capacity and increase vulnerability to occupational stress and depersonalization (67).

Adequate, high-quality sleep is essential for maintaining overall health, well-being, and optimal functioning. In contrast, insufficient sleep and untreated sleep disorders are linked to adverse physical and mental health outcomes, reduced quality of life, and increased risks to public safety (75). Accumulating evidence indicates that poor sleep quality is associated with higher burnout among healthcare professionals (76, 77). Nurses reporting poor sleep quality in this study showed higher odds of burnout than those with good sleep quality, consistent with findings from China (73) and a local study (13). During the COVID-19 pandemic, good sleep quality (PSQI ≤5) was associated with 5.7-fold higher odds of being free from burnout among frontline nurses (78). Sleep disruption and circadian misalignment have been linked to impaired emotional regulation, cognitive functioning, and resilience to stress, while night shifts and inadequate sleep have been associated with HPA axis dysregulation, altered cortisol and melatonin secretion, and chronic fatigue (25, 26), all of which may increase vulnerability to emotional exhaustion and poorer psychological well-being.

A substantial body of literature reports a strong association between burnout and depressive symptoms among healthcare workers (79). Individuals with depressive symptoms in this study showed higher odds of burnout, consistent with evidence from Ethiopia (14, 32) and a local study (13). However, the nature of this relationship remains debated. Some researchers argue that the moderate-to-high correlations between burnout and depression suggest that burnout may not represent a distinct psychological entity, but rather a context-specific manifestation of depression (80). Supporting this view, Schonfeld and Bianchi have proposed that burnout may largely reflect depressive pathology, with potential implications for depression-focused interventions (81). This interpretation is not universally accepted. Alternative perspectives emphasize that, despite overlapping features such as reduced energy and motivation, burnout and depression differ in phenomenology, etiology, and contextual specificity, with burnout more closely linked to chronic occupational stress (82). Furthermore, given the cross-sectional design of the present study, the temporal direction of the association cannot be determined. Longitudinal studies have suggested that depressive symptoms and burnout may exhibit reciprocal or bidirectional relationships over time, such that each may contribute to the development or persistence of the other. Therefore, depressive symptoms may contribute to burnout, burnout may contribute to depressive symptoms, or the relationship may be bidirectional (83, 84).

Anxiety has been identified as a factor associated with burnout syndrome, although it has received less attention than depression in the literature. Nurses reporting anxiety symptoms in this study showed higher odds of burnout than those without such symptoms, consistent with findings from Ethiopia (14), Korea (85), China (86), and a local study (13). Anxiety has been linked to heightened emotional arousal, impaired emotional regulation, and increased vulnerability to emotional exhaustion and depersonalization. Anxiety has been linked to heightened emotional arousal, impaired emotional regulation, dysregulation of the HPA axis, and sustained physiological stress responses (87, 88). Anxiety have also been associated with poorer sleep quality, reduced coping capacity, impaired concentration, and diminished occupational functioning (89). These factors may contribute to greater vulnerability to workplace stress and may help explain the association between anxiety symptoms and burnout observed in the present study. However, given the cross-sectional design of the present study, the temporal direction of this association cannot be determined. Anxiety symptoms may contribute to burnout, burnout may contribute to anxiety symptoms, or the relationship may be bidirectional (90). Longitudinal studies are needed to clarify the nature and direction of this relationship.

Taken together, the pattern of findings is broadly consistent with the JD-R framework (17, 18). High workload and mixed shifts represent substantial occupational demands, while longer service duration may reflect cumulative exposure to such demands. Job dissatisfaction may reflect limited occupational resources, whereas chronic illness, poor sleep, and psychological distress may compromise recovery and coping capacity. Notably, social support, an important resource within the JD-R framework (18), was associated with burnout in bivariable analysis but was no longer statistically significant after adjustment within the psychosocial model. Its attenuation may reflect overlap with other psychosocial factors, while job satisfaction remained associated with burnout, potentially reflecting its more direct relationship with the work environment. Monthly income likewise did not remain associated with burnout after adjustment within the sociodemographic/occupational model. Overall, the findings suggest that burnout among nurses in this setting may be understood in the context of coexisting occupational demands, available resources, and health- and recovery-related conditions rather than any single factor alone.

### Strengths and limitations

This study has several notable strengths. It provides one of the first empirical assessments of burnout syndrome among nurses in Mogadishu, offering baseline evidence from a largely under-researched, resource-limited context. The multicenter design, including both public and private hospitals, improved representation of diverse clinical settings and enhanced generalizability within the urban healthcare system. The use of standardized, validated instruments supported measurement reliability and comparability with international studies. In addition, robust statistical procedures—including assessment of model fit, multicollinearity, explanatory power, and discriminative performance—strengthened the rigor and transparency of the analysis.

Several limitations should be considered. The cross-sectional design precludes causal inference and limits conclusions on temporal relationships. Reliance on self-reported measures may have introduced recall and social-desirability bias, although anonymity and validated instruments likely reduced this risk. Convenience sampling from hospitals in Mogadishu may limit the representativeness of the sample and the generalizability of the findings beyond urban or hospital-based settings. Nurses who chose to participate may have differed systematically from those who did not participate with respect to burnout and related factors, introducing the possibility of selection bias. In addition, online data collection and WhatsApp-based recruitment may have excluded nurses with limited digital access, lower engagement with professional communication platforms, heavy workloads, or more severe burnout, which may have influenced participant selection and introduced selection bias. The relatively small number of burnout events (n = 53) limited the number of variables that could be included simultaneously in the multivariable analysis and may have reduced the stability and precision of some adjusted estimates, as reflected by the relatively wide confidence intervals. Separate sociodemographic/occupational and psychosocial models were therefore fitted to reduce model complexity and overfitting. Consequently, adjusted associations reflect within-model adjustment only, and residual confounding between domains cannot be excluded; findings should therefore be interpreted cautiously and confirmed in larger studies. Several brief psychosocial measures, particularly the PHQ-2, GAD-2, and OSSS-3, demonstrated low-to-moderate internal consistency in the current sample (Supplementary Table S1). Although coefficient alpha has limitations for very brief scales, these findings indicate potential measurement imprecision and possible misclassification of depressive symptoms, anxiety symptoms, and perceived social support. This is particularly relevant because depressive and anxiety symptoms remained associated with burnout in the adjusted psychosocial model. Accordingly, these associations should be interpreted with caution, especially given the absence of formal translation and cultural validation of these instruments in this population. Future studies with larger sample sizes should consider comprehensive multivariable models incorporating all relevant variables. Participants were recruited from five hospitals with unequal sample contributions, and potential within-hospital clustering was not accounted for. Unmeasured hospital-level characteristics may therefore have influenced the observed associations, and residual hospital-level confounding cannot be excluded. Finally, the absence of formal translation and cross-cultural validation of the English-language instruments may have affected measurement equivalence and participants’ interpretation of individual items, particularly for measures demonstrating relatively low internal consistency in the current sample. Consequently, findings based on these measures should be interpreted with caution, and future research should prioritize formally validated and culturally adapted Somali versions.

### Practical and policy implications

These findings underscore the need for coordinated workplace and policy responses to burnout among nurses in Somali healthcare settings. At the institutional level, efforts should focus on optimizing staffing, managing workload, and improving shift scheduling, particularly by limiting prolonged or mixed day–night duties where feasible. Supportive supervision, recognition of staff contributions, and opportunities for professional development may also strengthen job satisfaction and workplace resilience.

Occupational health services should consider incorporating routine screening for burnout, depressive and anxiety symptoms, and sleep problems to facilitate early identification of nurses who may require additional support. Ensuring adequate rest periods and access to appropriate mental health and staff-support services may further promote well-being and functioning.

At the policy level, health authorities and hospital leadership should work toward workforce standards that promote safe staffing, supportive working environments, and integration of staff mental health into occupational health planning. Such measures may contribute to improved nurse well-being and, potentially, the quality and continuity of patient care in resource-limited settings.

## Conclusion and recommendations

This study provides one of the first systematic assessments of burnout among nurses in Mogadishu, with 18.2% meeting the study definition of burnout. Higher odds of burnout were observed among nurses with high workload, mixed shifts, longer service duration, chronic illness, job dissatisfaction, depressive and anxiety symptoms, and poor sleep quality. These findings highlight the multifactorial nature of burnout and the importance of considering both occupational and psychosocial factors when addressing nurse well-being.

From a research perspective, the study provides baseline evidence from a resource-limited setting and addresses an important gap in the literature. Longitudinal studies are needed to clarify temporal relationships between burnout and associated factors, while interventional research should evaluate the effectiveness of strategies aimed at reducing burnout and improving nurse well-being in similar settings.

## Data Availability

The raw data supporting the conclusions of this article will be made available by the authors, without undue reservation.
