# Supplementary material for: Prevalence of burnout syndrome and associated factors among nurses in Mogadishu, Somalia: a multicenter cross-sectional study
Source: Front Public Health. 2026 Oct 1;14:1951920. doi: 10.3389/fpubh.2026.1951920 (PMC13668534; doi:10.3389/fpubh.2026.1951920)
Supplement: Supplementary file 1 [file Table_1.docx]

**Supplementary Table**

**Supplementary Table S1. Internal consistency of multi-item study instruments in the current sample**

| **Instrument/subscale** | **No. of items** | **Cronbach’s α** |
| --- | --- | --- |
| Emotional Exhaustion (EE) | 9 | 0.84 |
| Depersonalization (DP) | 5 | 0.72 |
| Personal Accomplishment (PA) | 8 | 0.79 |
| PHQ-2 | 2 | 0.40 |
| GAD-2 | 2 | 0.60 |
| OSSS-3 | 3 | 0.59 |

**Note:** EE, Emotional Exhaustion; DP, Depersonalization; PA, Personal Accomplishment; PHQ-2, Patient Health Questionnaire-2; GAD-2, Generalized Anxiety Disorder-2; OSSS-3, Oslo Social Support Scale-3.

**Supplementary Table S2. Bivariable logistic regression analysis of factors associated with burnout among nurses**

| **Variable** | **Category** | **Crude OR (95% CI)** | ***p*-value** | **Overall *p*-value** |
| --- | --- | --- | --- | --- |
| Age | 18–29 years | 1.28 (0.63–2.58) | 0.497 | 0.497 |
|  | ≥ 30 years | 1 | 1 |  |
| Biological sex | Male | 1 | 1 | 0.503 |
|  | Female | 1.25 (0.65–2.41) | 0.503 |  |
| Marital status | Unmarried | 0.74 (0.39–1.43) | 0.371 | 0.371 |
|  | Married | 1 | 1 |  |
| Monthly income | ≤600 USD | 1.68 (0.80–3.52) | 0.174 | 0.174 |
|  | >600 USD | 1 | 1 |  |
| Education status | Diploma | 2.08 (0.76–5.70) | 0.154 | 0.346 |
|  | Bachelor degree | 1.08 (0.57–2.06) | 0.809 |  |
|  | Master’s and above | 1 | 1 |  |
| Chronic medical condition | Yes | 3.75 (1.97–7.17) | <0.001 | <0.001 |
|  | No | 1 | 1 |  |
| Physical activity | Yes | 1 | 1 | 0.511 |
|  | No | 0.66 (0.19–2.30) | 0.511 |  |
| Current substance use | Yes | 1.40 (0.77–2.55) | 0.269 | 0.269 |
|  | No | 1 | 1 |  |
| Job title | Nurse administrator | 1 | 1 | 0.309 |
|  | Registered nurse (RN) | 0.68 (0.32–1.43) | 0.309 |  |
| Work shift | Regular day shift | 1 | 1 | 0.031 |
|  | Regular night shift | 1.32 (0.49–3.55) | 0.583 |  |
|  | Mixed shifts | 2.46 (1.22–4.96) | 0.012 |  |
| Work hours per day | ≤ 10 hours | 1 | 1 | 0.465 |
|  | > 10 hours | 1.25 (0.69–2.27) | 0.465 |  |
| Years of experience | < 5 years | 1 | 1 | <0.001 |
|  | 5–10 years | 0.47 (0.22–1.01) | 0.053 |  |
|  | > 10 years | 3.43 (1.46–8.06) | 0.005 |  |
| Institutional affiliation | Erdogan hospital | 1.01 (0.42–2.42) | 0.978 | 0.359 |
|  | Hodan hospital | 0.89 (0.28–2.84) | 0.845 |  |
|  | Shaafi hospital | 0.68 (0.19–2.48) | 0.557 |  |
|  | Kalkaal hospital | 2.25 (0.75–6.76) | 0.149 |  |
|  | Mogadishu specialist hospital | 1 | 1 |  |
| Job satisfaction | Satisfied | 1 | 1 | 0.002 |
|  | Dissatisfied | 3.37 (1.56–7.28) | 0.002 |  |
| Workload | Yes | 3.56 (1.54–8.24) | 0.003 | 0.003 |
|  | No | 1 | 1 |  |
| Depression symptoms | Absent | 1 | 1 | <0.001 |
|  | Present | 7.11 (3.74–13.53) | <0.001 |  |
| Anxiety symptoms | Absent | 1 | 1 | <0.001 |
|  | Present | 6.81 (3.57–13.01) | <0.001 |  |
| Sleep quality | Poor | 2.23 (1.17–4.28) | 0.015 | 0.015 |
|  | Good | 1 | 1 |  |
| Social support | Poor | 3.67 (1.23–10.91) | 0.019 | <0.001 |
|  | Moderate | 0.86 (0.24–3.01) | 0.810 |  |
|  | High | 1 |  |  |

**Note:** OR, odds ratio; CI, confidence interval. For multicategorical variables, the p-value in the Overall row represents the overall Wald χ² test; category-specific crude ORs are relative to the reference category.
